# Pneumococcal Disease Prevention: Are We on the Right Track?

**DOI:** 10.3390/vaccines9040305

**Published:** 2021-03-24

**Authors:** Nicola Principi, Susanna Esposito

**Affiliations:** 1 Università Degli Studi di Milano, 20122 Milan, Italy; nicola.principi@unimi.it; 2 Pediatric Clinic, Pietro Barilla Children’s Hospital, University of Parma, 43126 Parma, Italy

**Keywords:** invasive pneumococcal diseases, pneumococcal conjugate vaccine, protein vaccine, serotype 3, *Streptococcus pneumoniae*

## Abstract

The history of *Streptococcus* pneumoniae diseases dramatically changed with the introduction into the immunization schedule of infants and children of the first pneumococcal conjugate vaccine, the one containing 7 (PCV7) of the most common pneumococcal serotypes (STs) causing invasive pneumococcal diseases (IPDs). Where PCV7 was largely used, incidence of both IPDs and non-invasive pneumococcal diseases (nIPDs) in vaccinated children and in unvaccinated subjects of any age, mainly the elderly, significantly decreased. Unfortunately, the impact of PCV7 administration was slightly lower than expected, as the reduction in infections due to vaccine serotypes (STs) was accompanied by a significant increase in the number of IPDs and nIPDs due to STs not included in the vaccine. To overcome this problem, two PCVs containing 10 (PCV10) and 13 (PCV13) STs, chosen among those emerging, were developed and licensed. However, ST replacement occurred again. Moreover, the new PCVs showed little effectiveness in the prevention of infection due to non-encapsulated STs and to ST3. Next-generation *S. pneumoniae* vaccines able to prevent pneumococcal infections regardless of infecting ST are urgently needed. For the moment, the use of available PCVs remains fundamental because their benefits far outweigh any concerns for emerging STs.

## 1. Background

The history of *Streptococcus pneumoniae* diseases dramatically changed at the beginning of this century with the introduction into the immunization schedule of infants and children of the first pneumococcal conjugate vaccine, a preparation containing 7 (PCV7) of the most common pneumococcal serotypes (STs) causing invasive pneumococcal diseases (IPDs). Where PCV7 was largely used, incidence of both IPDs and non-invasive pneumococcal diseases (nIPDs) in vaccinated children and in unvaccinated subjects of any age, mainly the elderly, significantly decreased. Enormous medical, social, and economic advantages were achieved [1].

## 2. From the 7-Valent Pneumococcal Conjugate Vaccine (PCV7) to the 10-Valent (PCV10) and 13-Valent (PCV13) Vaccines

Despite the benefits of PCV7 administration, an accurate analysis of IPD epidemiology after PCV7 introduction showed that the reduction in IPD incidence was slightly lower than expected, as the decline in the number of infections due to vaccine STs was accompanied by a significant increase in the number of IPDs and nIPDs due to STs not included in the vaccine [2,3]. To overcome this problem, two PCVs containing 3 (PCV10) and 6 (PCV13) more STs, chosen among those emerging, were developed and licensed roughly 10 years after PCV7 introduction. They substituted PCV7 but ST replacement occurred again, showing that new vaccines with a greater number of serotypes were needed to face the continuously emerging infections [4,5].

Two vaccines containing 15 [6] and 20 STs [7] are in advanced development and were found immunogenic and safe. However, even if they were licensed for use in humans, it seems highly unlikely that they can definitively solve the problem of *S. pneumoniae* infection prevention. Approximately 100 pneumococcal STs have been identified and replacement can occur again, reducing the efficacy of the new vaccines. On the other hand, a further increase in the number of STs in PCV formulation does not seem feasible as the production of PCV vaccines becomes more complex, problematic, and expensive with the addition of more STs in each preparation. This explains why, in order to have broader ST coverage, new approaches to pneumococcal vaccine development have been undertaken in recent years. Preparations based on conserved pneumococcal proteins, killed whole-cell vaccines, and live attenuated whole-cell vaccines have been studied. Delivery systems capable of increasing immune stimulation have been developed. In some cases, results are promising and suggest that this could be the right way to overcome the problems of PCVs [8]. An example in this regard is given by the use of LytB protein, a well-conserved, surface-exposed protein of *S. pneumoniae* involved in different aspects of the pathogenesis process. Immunization of mice with this protein induced protection against invasive pneumonia and sepsis due to different pneumococcal STs, including ST3 [9]. Finally, a further advantage for protection against *S. pneumoniae* infections could be obtained by the use, at least in those patients for whom they are recommended, of alternative vaccines able to exert heterologous protection against pneumococcal strains, including ST3. Tarancon et al. [10]. have shown that the administration of MTBVAC, a live attenuated strain of Mycobacterium tuberculosis in phase III as a vaccine candidate, can produce metabolic and epigenetic reprogramming of human monocytes and trained immunity in vivo, leading to protection against experimental CAP due to ST3.

## 3. Limitations of Pneumococcal Prevention

The need for new pneumococcal vaccines is further demonstrated by the evidence that PCVs, including the newest, cannot prevent infections due to non-encapsulated (NE) *S. pneumoniae*. Although, generally, NE *S. pneumoniae* causes nIPDs such as conjunctivitis and otitis media, the risk that they are associated with in the development of IPDs, particularly in immunocompromised subjects, cannot be excluded. Moreover, NE *S. pneumoniae* could play a relevant role in the diffusion of antibiotic resistance and virulence genes as these strains have been found to be able to acquire genes more frequently than capsulated bacteria [11].

However, the most important limitation of PCVs is the low or lack of effectiveness in the prevention of infection due to ST3. This is a very important problem as ST3 is frequently associated with a number of very severe diseases, including meningitis, bacteremia-associated septic shock, bacteremic and complicated pneumonia, and, as recently reported, it may play a role in conditioning adverse cardiac events in hospitalized patients [12]. Despite this evidence, ST3 was not included in PCV10 formulation by the manufacturer of this vaccine because studies conducted during the initial phase of development had shown that the presence of ST3 could not prevent ST3 diseases [8]. Moreover, rates of prevention of ST3 diseases following extensive PCV13 use were generally poorly satisfactory. Although a certain degree of protection was demonstrated in some studies in both children and adults [13,14], in most of cases, the reduction in ST3 infections after PCV13 was lower than that shown for all the other STs included in this vaccine. In Spain, analysis of the nationwide trends of IPDs from 2009 through 2019 has shown that, whereas the global reduction in IPDs due to PCV13 STs in children was close to 90% for both children <2 years and children between 2 and 5 years, reduction in IPDs due to ST3 was lower than 40%. [15]. In the United Kingdom, a long-term evaluation of PCV efficacy against IPD [16] showed that, 4 years after PCV13 introduction, the incidence of ST3 IPDs remained substantial and the vaccine efficacy against this ST was significantly lower than that offered against all the other STs. In children 2–9 years of age who had received the 2 + 1 dose immunization schedule, vaccine was even negative as efficacy was −141% (95% confidence interval (CI) −700 to 17.3) and the total number of ST3 IPDs, compared with the previous period, was increased. Similar findings were reported in Spain [17], Denmark [18], and Japan [19].

The best evidence of the poor effect of PCV13 against ST3 infections was collected when pneumonia, mainly the most complicated cases, was evaluated. A study regarding the incidence and etiology of necrotizing pneumonia among Italian children revealed that, 6 years after PCV13 introduction, the incidence of this disease tended to increase and ST3 was the main etiologic agent both before and after PCV13 use [20]. A predominance of empyema due to ST3 following the introduction of PCV13 has been documented in Germany [21] and Greece [22]. Finally, a very recent study carried out in Australia [23] showed that 76.4% of STs identified in empyema cases after PCV13 introduction were ST3, a value more than double than that calculated during the PCV7 period.

Reasons for the poor efficacy of PCV13 against ST3 infections are not precisely defined. Several mechanisms have been suggested [24]. Among them, the most important seems the ability of ST3 to produce and release a greater amount of capsular polysaccharide than the other STs. The disproportion between the quantity of one of the most important contributors to *S. pneumoniae* virulence and the quantity of antibodies evoked by the PCV13 for its neutralization is considered the main cause of the poor efficacy of PCV13 against ST3 infections. Several studies support this conclusion. In vitro, it has been shown that the released capsular polysaccharide concentrations were greater in ST3 culture than in ST1, ST4, ST6B, and ST14 cultures [25]. In experimental animal models, it has been demonstrated that a marginal amount of ST3 culture supernatant could inhibit antibody protection, whereas, when other STs were tested, concentrations more than 100 times higher were required for the inhibition of protection [18]. In humans, evidence of the insufficient production of ST3-neutralizing antibodies by PCV13 has been reported in a study [26] in which it was calculated that the serum IgG concentration needed to prevent ST3 IPDs was significantly higher than that usually attained from vaccination. For most STs, a specific serum antibody concentration of 0.35 µg/mL was found sufficient to prevent IPDs, whereas a value of 2.83 µg/mL was calculated for ST3.

Final support for the development of new pneumococcal vaccines is given by the evidence that neither the 15- nor the 20-valent conjugate vaccines seem able to significantly improve protection against ST3 infections. The 20-valent vaccine is produced by the same manufacturer of PCV13 and does not differ in ST3 content. It has been reported that the 15-valent vaccine evokes a more sustained antibody response against ST3 than PCV13 [6]. However, analysis of the results of the study carried out in infants receiving the four-dose immunization schedule reveals that antibody concentrations achieved after PCV15, despite being higher than those obtained with PCV13 after both the primary series and the booster dose, remain much lower than the putative correlate of protection.

## 4. Conclusions

Next-generation *S. pneumoniae* vaccines able to confer protection against ST3 infection are urgently needed. Preparations with conserved pneumococcal proteins have been shown to protect experimental animal against IPDs due to ST3 [9,27]. However, we are still a long way from having an effective vaccine available against all *S. pneumoniae* infections. For the moment, let us be happy with what is available by continuing to vaccinate with current PCVs. Their use remains fundamental, although they have some limitations, which, in any case, remain far less than the great advantages that they have.
